# Protective Effects of *Lactobacillus rhamnosus* GG Against Methotrexate-Induced Oxidative Renal Toxicity

**DOI:** 10.1007/s12602-026-11052-4

**Published:** 2026-05-19

**Authors:** Bade Almurad, Bertan Boran Bayrak, Muhammet Oguzhan Donmez, Goksel Sener, Refiye Yanardag

**Affiliations:** 1 Department of Chemistry, Faculty of Engineering, Istanbul University-Cerrahpaşa, Istanbul, Türkiye; 2 Department of Pharmacology, Faculty of Pharmacy, Fenerbahçe University, Istanbul, Türkiye

**Keywords:** Methotrexate, *Lactobacillus rhamnosus* GG, Oxidative Stress, Renal Toxicity

## Abstract

Methotrexate (MTX) is commonly prescribed for various malignant and autoimmune conditions, but it can cause significant oxidative and functional impairment in renal tissue. *Lactobacillus rhamnosus* GG. (LGG) is a well-known probiotic with biological activities that support antioxidant balance. This study investigated the impact of LGG on MTX-induced kidney damage. Male Sprague–Dawley rats were divided into four groups: physiological saline-treated control group; a group receiving MTX alone; a group receiving MTX alongside a low dose of LGG; and a group receiving MTX alongside a high dose of LGG. MTX was administered as single dose (20 mg/kg/bw) intraperitoneally and LGG (low dose 1 x 10^9^ CFU/day and high dose 5 × 10^9^ CFU/day, respectively) orally for five days. On day six, blood and kidney samples were collected and examined for oxidative indicators, enzymatic antioxidant responses, and renal functional markers. MTX significantly increased in glomerular filtration markers in serum and elevated key indicators of oxidative stress in renal tissues. More so, MTX demonstrated to disrupt renal ionic homeostasis, such as declined sodium/potassium-ATPase, paraoxonase, and increased lactate dehydrogenase, carbonic anhydrase, xanthine oxidase, myeloperoxidase, and arginase activities. In contrast, LGG supplementation has been shown to effectively reverse all MTX-induced biochemical alterations in both serum and renal tissue. We can suggest that LGG can provide significant protection against oxidative renal toxicity induced by MTX in rats.

## Introduction

Methotrexate (MTX) is a widely used antifolate chemotherapeutic agent administered for various malignant and autoimmune diseases. Although effective, MTX is strongly associated with dose-dependent toxicities, particularly acute kidney injury (AKI), which occurs in approximately 2–12% of treated patients [1]. Because nearly 90% of MTX is eliminated through the kidneys, renal impairment markedly reduces MTX clearance and exacerbates systemic toxicity [2]. The primary mechanisms underlying methotrexate-induced nephrotoxicity include MTX’s very poor solubility at acidic pH, and the fact that its metabolites (i.e., 7-hydroxyMTX) are six to ten times less soluble than MTX itself. This results in intratubular crystal precipitation and direct tubular epithelial damage, both of which contribute to oxidative stress (OS), inflammation and impaired kidney function [3, 4]. Despite existing preventive strategies, MTX-associated AKI remains a significant clinical challenge, emphasizing the need for alternative protective approaches [5]. OS is defined as an imbalance between the production of reactive oxygen species (ROS) and the body’s antioxidant defense systems [6]. Excessive ROS can damage cellular lipids, proteins and DNA, resulting in structural and functional alterations to tissues [7]. In the kidneys, for example, elevated OS contributes to tubular injury, inflammation and impaired filtration capacity, making it a key pathogenic mechanism in the development and progression of acute and chronic kidney dysfunction [8].

*Lactobacillus rhamnosus* GG. (LGG) is a well-characterized probiotic strain that is widely recognized for its potent antioxidant and anti-inflammatory properties [9]. Recent studies have demonstrated that LGG can bolster cellular antioxidant defenses by increasing the production of enzymes such as superoxide dismutase (SOD) and glutathione peroxidase (GPx), while simultaneously curbing the production of reactive oxygen species [10]. Furthermore, LGG has been reported to stabilize epithelial barrier integrity and protect tissues from oxidative and inflammatory injury, contributing to improved cellular resilience and overall tissue homeostasis [11]. Moreover, experimental models have demonstrated that LGG can protect tissues, particularly hepatic and intestinal tissues, from chemically induced oxidative damage by reducing lipid peroxidation and improving antioxidant status [12]. Although LGG exhibits significant antioxidants and protective properties, its potential in preventing MTX-induced oxidative kidney injury remains unclear. Therefore, this study aimed to investigate whether LGG supplementation could alleviate MTX-induced kidney damage by evaluating serum parameters as an index of glomerular filtration, antioxidant enzyme activities in kidney tissue, and changes in kidney function parameters in a Sprague-Dawley rat model.

## Materials and Methods

### Chemicals

All chemicals used in this study were of analytical grade and purchased from Merck, Sigma, Aldrich, Riedel-de Haën, and/or Fluka. LGG was supplied by Menarini İlaç (Kaleidon) Istanbul, Türkiye.

## Experimental Animals and Grouping

This study was approved by the Marmara University Local Ethics Committee for Animal Experiments (Approval No: 6.2025mar7, dated 29.05.2025). Thirty-two male Sprague–Dawley rats (2–2.5 months old) were used. Before the experiments began, the rats were acclimated to environmental conditions in a 12-hour light/12-hour dark cycle and at a constant room temperature. Throughout the experiment, the animals were housed in polypropylene cages with free access to tap water and fed a standard pellet diet.

The rats were randomly assigned into four groups as follows:

### Control Group (*n* = 8)

To match the procedures used in the other groups, the control group received a single intraperitoneal injection of physiological saline and oral physiological saline for 5 days.

### MTX Group (*n* = 8)

Received a single intraperitoneal dose of MTX (Kocak Farma Pharmaceutical and Chemical Industry Co., Ltd. Turkey; 20 mg/kg ip) and oral physiological saline (0.1 mL/100 g body weight) for 5 days [13],

### MTX + LGG1 Group (*n* = 8)

Received a single intraperitoneal injection of MTX (20 mg/kg), followed by oral LGG (1 × 10⁹ CFU/day) for 5 consecutive days [14],

### MTX + LGG5 Goup (*n* = 8)

Received a single intraperitoneal injection of MTX (20 mg/kg), followed by oral LGG (5 × 10⁹ CFU/day) for 5 consecutive days.

LGG dosing was based on the declared CFU content of the preparation. The suspension was prepared freshly before oral administration. However, bacterial viability, formulation stability, and actual administered CFU were not independently verified during the study period.

**Fig. 1:**
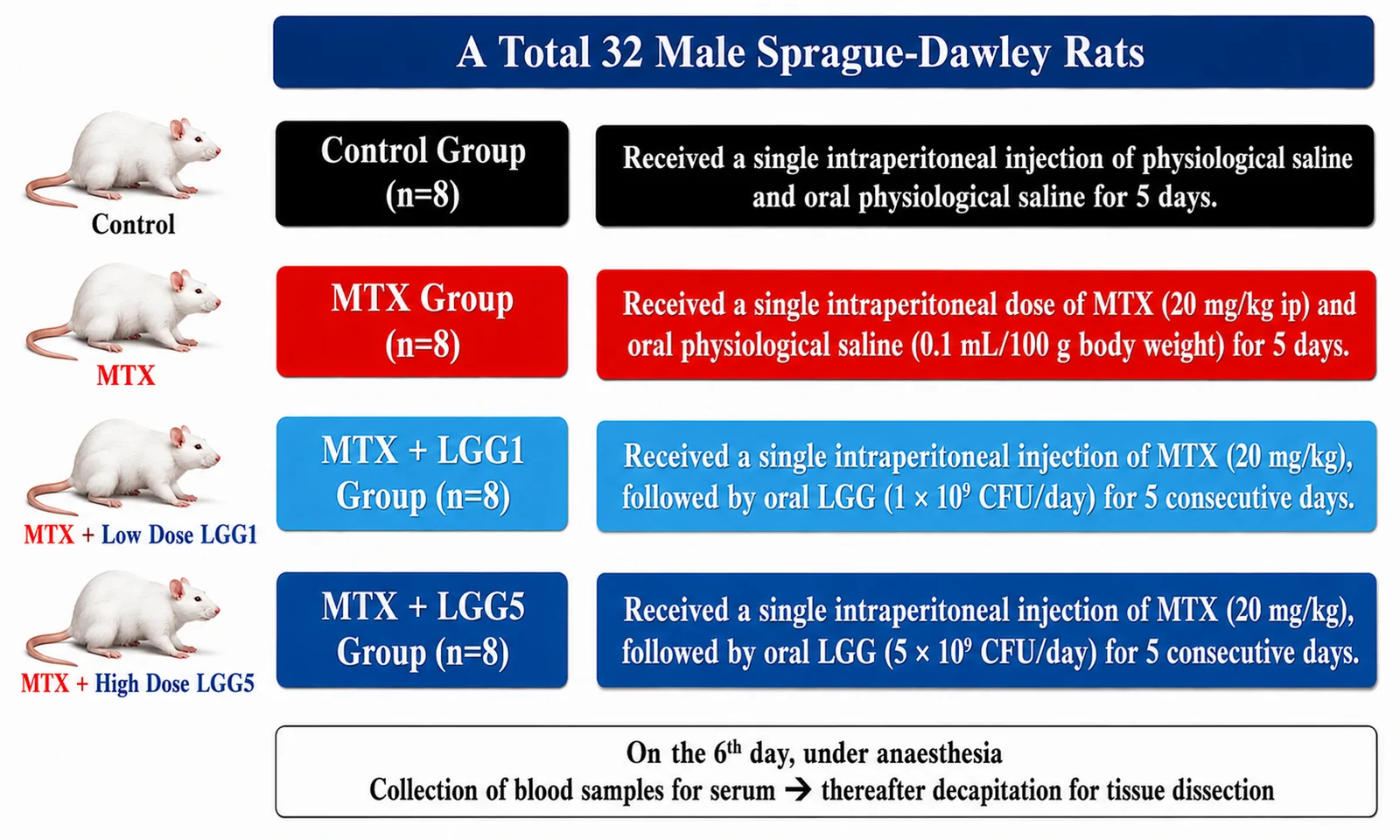
Schematic representation of experimental design

Following the experimental period at the end of the experiments (on the sixth day), the animals were placed in a state of fasting overnight. After blood samples were collected from the hearts of all rats under anesthesia using sterile syringes, the rats were decapitated. Thereafter, their kidney tissues were harvested, weighed, and immediately immersed in chilled physiological saline and frozen at − 76 °C. Schematic representation of experimental design was depicted in Fig. 1.

## Preparation of Serum Samples

Blood samples were centrifuged at 3000 rpm for 15 min, and the serum was separated and stored at − 76 °C until analysis. Before assays, samples were thawed overnight at − 20 °C and then kept at + 4 °C for one additional night.

## Preparation of Kidney Tissue Homogenates

Prior to homogenization, tissues kept in −76 °C were thawed at −20 °C overnight and kept at + 4 °C the following night. After blotting on filter paper, each tissue was weighed and homogenized in ice-cold physiological saline to prepare 10% (weight/volume) homogenates using a glass homogenizer. Homogenates were centrifuged at 10.000 rpm (~ 8832 x g) for 20 min at + 4 °C, and the supernatants collected were stored at − 76 °C for biochemical analyses.

## Biochemical Analysis

### Determination of Serum Urea Levels

The urea level in the serum was determined using Barker’s method [15]. In brief, the urea content was quantified colorimetrically using the diethyl monoxime reaction. The deproteinized tissue homogenate was mixed with the oxime reagent in an acidic medium and boiled for 15 min. Once cooled, the absorbance of the resulting mixture was measured using spectrophotometer (Shimadzu UV-Mini-1240, Kyoto, Japan) at a wavelength of 520 nm. The results were reported as mmol/L.

## Determination of Serum Creatinine Levels

The creatinine level in the serum was assessed by the protocol of Bonsnes and Taussky [16]. Briefly, the deproteinized tissue homogenate was mixed with the alkaline picric acid reagent and allowed to stand in the dark at room temperature for 20 min. The resulting mixture was then measured spectrophotometrically for absorbance at a wavelength of 520 nm. The results were reported as µmol/L.

## Determination of Serum Uric Acid Levels

The uric acid level in the serum was quantified by the Caraway’s method [17], which is based on the reduction of phosphotungstate by uric acid in alkaline solution. In brief, the deproteinized tissue sample was mixed with the Folin-Denis reagent (phosphotungstic acid solution) and allowed to stand in the dark at room temperature for 20 min. Following the incubation period, the absorbance of the blue-colored product produced by the reduction of phosphotungstate was measured at 700 nm using a spectrophotometer. The results were reported as µg/dL.

### Determination of Serum Urea/Creatinine Ratio Levels

In order to calculate serum urea/creatinine ratio (UCR) levels the following equation was used [18]:

UCR = [Serum urea (mmol/L)/(plasma creatinine (µmol/L) divided by 1000)]

### Determination of Kidney Reduced Glutathione Levels

The reduced glutathione (GSH) levels were assayed according to the method of Beutler [19] using Ellman’s reagent based on the formation of a yellow-colored complex with 5,5-dithiobis-(2-nitrobenzoic acid) (DTNB) and measured spectrophotometrically at 412 nm. The results were expressed as nmol GSH/mg protein.

### Determination of Kidney Glutathione Peroxidase Activities

The glutathione peroxidase (GPx) activities in kidney homogenate were estimated by the kinetic reaction protocol developed by Paglia and Valentine [20]. GPx catalyzes the oxidation of GSH in the presence of H_2_O_2_. As a result, oxidized glutathione (GSSG) and H_2_O are formed. The resulting GSSG is reduced back to GSH by a reaction catalyzed by glutathione reductase (GR) using NADPH as a reducing coenzyme. During these reactions, the oxidation of the coenzyme leads to a decrease in the absorbance value. GPx activity is calculated by measuring the change in absorbance at a wavelength of 366 nm per minute for 5 min with a spectrophotometer. The specific activity of GPx was expressed as U/g protein.

### Determination of Kidney Glutathione Reductase Activities

The activities of glutathione reductase of kidney tissues of rats were estimated by the kinetic reaction protocol developed by Beutler [21]. Briefly, reaction mixture containing Tris-HCl buffer (pH 8.0), NADPH, and GSSG were mixed with tissue samples. The decline in absorbance because of oxidation of NADPH was recorded in a spectrophotometer at 340 nm per minute for 5 min. The specific activity of GR was expressed as U/g protein.

### Determination of Kidney Glutathione-S-Transferase Activities

The glutathione-S-transferase (GST) activity was determined according to the method of Habig and Jakoby [22]. Briefly, potassium phosphate buffer (pH 6.6), appropriate volume of homogenate, GSH, 1-chloro-2,4-dinitrobenzene (CDNB, in absolute ethanol), and distilled water were mixed. The increase in absorbance of the mixture was observed at room temperature at a wavelength of 340 nm per minute for 3 min. The specific activity of GST was expressed as U/g protein.

### Determination of Kidney Lipid Peroxidation Levels

The lipid peroxidation (LPO) levels were estimated according to Ledwozyw et al. [23]. Briefly, tissue homogenate and trichloroacetic acid solution were mixed and allowed to stand at room temperature for 15 min. The thiobarbituric acid solution was added to the reaction medium and then boiled in a water bath at 95 °C for 30 min. The resultant mixture was mixed with the n-butanol solution to extract organic phase. The absorbance of the organic phase at 532 nm was then recorded using a spectrophotometer in terms of kidney malondialdehyde (MDA), which is undertaken as an index of LPO. The results were expressed as nmol MDA/mg protein.

### Determination of Kidney Advanced Oxidized Protein Products Levels

The levels of advanced oxidized protein products (AOPP) were determined by means of the Witko–Sarsat protocol [24]. In summary, the diluted tissue homogenate and phosphate buffer (pH 7.4) solution were mixed with the KI solution and then left to incubate at room temperature for a period of two minutes. Subsequently, the solution was transferred into a cuvette and mixed with concentrated acetic acid. Absorbance was measured at a wavelength of 340 nm. The results were reported as pmol/mg protein.

### Determination of Kidney Reactive Oxygen Species Levels

The levels of reactive oxygen species (ROS) were measured using a fluorometric method, with dichlorofluorescein-diacetate (DCFH-DA) serving as the fluorescent probe using BioTek FLx800 fluorescence microplate reader. The excitation and emission wavelengths used were 480 and 535 nm, respectively [25]. The results were reported as arbitrary fluorescence unit (AFU)/mg protein.

### Determination of Kidney Superoxide Dismutase Activities

The superoxidase dismutase (SOD) activity was assessed by using the method developed by Mylroie et al. [26]. The catalytic activity of the SOD enzyme is determined by measuring the ability of riboflavin-sensitized o-dianisidine to increase the photooxidation rate under UV light. The superoxide radical formed by riboflavin under the influence of UV light is converted into H_2_O_2_ by the catalytic effect of the SOD. The first absorbance value of the reaction initiated by the addition of riboflavin was measured in a spectrophotometer at 460 nm. The second absorbance of the samples, which were kept under UV light for 8 min, was measured again. The difference between the absorbance values obtained at 0 and 8 min was taken, and the specific activity of SOD was expressed as U/mg protein.

### Determination of Kidney Catalase Activities

The catalase (CAT) activity was measured using the method developed by Aebi [27]; the decrease in absorbance (between 0 and 1 min) accompanying the reduction of H₂O₂ to H₂O in the presence of CAT was read spectrophotometrically at 240 nm. The specific activity of CAT was defined as U/mg protein.

### Determination of Kidney Lactate Dehydrogenase Activities

The lactate dehydrogenase (LDH) activity was determined using the method described by Bais and Philcox [28]. In brief, the kidney homogenate was mixed with a solution of NADH (dissolved in Tris-HCl buffer at pH 7.4) and then incubated at 37 °C for five minutes. The resultant mixture was then supplemented with a substrate solution (pyruvate). The oxidation of NADH to NAD⁺ was then recorded at 340 nm. The specific activity of LDH was defined as AFU/µg protein.

### Determination of Kidney Paraoxonase Activities

The activities of paraoxonase (PON) in kidney homogenates were evaluated according to the methodology outlined by Furlong et al. [29]. Spectrophotometric analysis was conducted in a Tris-HCl buffer (pH 8.5) using paraoxon-ethyl as the substrate. The analysis was performed at a temperature of 37 °C, and the wavelength of 405 nm was utilized for the measurement. The specific activity of PON was defined as U/g protein.

#### Determination of Kidney Myeloperoxidase Activities

The activities of myeloperoxidase (MPO) in kidney homogenates were assessed according to the methodology outlined by Wei and Frenkel [30]. To this end, the 4-aminoantipyrine/phenol substrate was added to the H₂O₂ solution, after which the mixture was left to stand for 3–4 min to reach equilibrium. The tissue homogenate was then added to the resulting mixture and the increase in absorbance at 510 nm was recorded per minute for 5 min. The specific activity of MPO was expressed as U/g tissue.

### Determination of Kidney Xanthine Oxidase Activities

The xanthine oxidase (XO) activity was measured according to Corte and Stirpe’s protocol [31]. In brief, a phosphate buffer solution (pH 7.4) containing EDTA and xanthine (the substrate) was added to the tissue homogenate, and the mixture was incubated for 10 min at 24 °C. After the incubation period, the formation of uric acid was monitored at 280 nm. The specific activity of XO was expressed as U/g protein.

#### Determination of Kidney Sodium/Potassium-ATPase Activities

The sodium/potassium-ATPase (Na^+^/K^+^-ATPase) activity was determined according to the method of Ridderstap and Bonting [32] by measuring inorganic phosphate released from ATP hydrolysis at 700 nm. To do this, the homogenates were incubated with the appropriate amount of ATP. First, total ATPase activities were assayed thereafter Mg^2+^-ATPase activities were determined in the presence of ouabain. The Na^+^/K^+^-ATPase activities were calculated by subtraction from the total ATPase of Mg^2+^-ATPase. The specific activity of Na^+^/K^+^-ATPase was expressed as µmol P*i*/mg protein/h.

#### Determination of Kidney Carbonic Anhydrase Activities

The carbonic anhydrase (CA) activities in the kidney tissue homogenates were evaluated the method developed by Verpoorte et al. [33]. This method is essentially based on the esterase activity of CA. Depending on the pH of the medium, the CA enzyme catalyzes the hydrolysis of p-nitrophenyl acetate, which acts as a substrate, to p-nitrophenol. To this end, a Tris-SO₄ buffer (pH 7.4) is mixed with the kidney homogenate. The substrate is then added to the cuvette. The absorbance of the p-nitrophenol mixture, which is formed as a result of CA activity, is measured spectrophotometrically at 348 nm per minute for 5 min. The specific activity of CA was expressed as U/mg protein.

#### Determination of Kidney Arginase Activities

The arginase activities were determined by the protocol of Geyer and Dabich [34]. The samples were subjected to an initial incubation period of 5 min at a temperature of 65 °C, in the presence of MnCl₂. This was followed by a subsequent incubation period of 10 min at a temperature of 37 °C, in the presence of L-arginine. The reaction was stopped by the addition of diacetyl monoxime reagent (containing thiosemicarbazide) following acid reagent (containing FeCl_3_ in concentrated H_3_PO_4_). Subsequently, the samples were subjected to boiling water for a period of 10 min. The urea concentration, indicative of arginase activity, was measured spectrophotometrically at a wavelength of 520 nm. The specific activity of arginase was expressed µmol urea/mg protein at a temperature of 37 °C.

### Determination of Kidney Nitric Oxide Levels

The nitric oxide (NO) levels were carried out by spectrophotometric method of Miranda et al. [35]. The principle of this assay is reduction of nitrate to nitrite by VCl_3_ in an acidic reaction medium containing Griess reagent [mixed an equal volume of sulfanilamide (2% w/v) and N-(1-Naphtyl) ethylenediamine dihydrochloride (0.1% w/v)]. After the colored diazonium complex completed, absorbance of the resultant mixture was measured at 540 nm by a spectrophotometer, and the results were expressed as nmol NO/mg protein.

### Determination of Kidney Hydroxyproline Levels

The hydroxyproline (Hypro) levels were determined by the method of Reddy and Enwemeka [36]. This assay is based on alkaline hydrolysis of tissue homogenates thereafter subsequent determination of free hydroxyproline content in hydrolysates. After the hydrolyzation process was done at 110 °C for 3 h, hydrolysates were mixed with chloramine-T solution and the oxidation of free hydroxyproline contents was allowed to proceed for 25 min at room temperature. Ehrlich’s reagent [p-dimethylaminobenzaldehyde in n-propanol/perchloric acid (2:1 ratio v/v) solution] was added to reaction medium and the chromophore was developed by incubating the samples at 65 °C for 20 min. The absorbance of the samples was then recorded at 550 nm using a spectrophotometer. The results were expressed as µg Hypro/mg protein.

## Determination of Kidney Total Protein Contents

Total protein levels in the homogenates were estimated according to the method of Lowry et al. [37]. Briefly, proteins were reacted with Cu^2+^ ions in alkaline medium and reduced by the Folin-Ciocalteu reagent. The absorbance of the blue-colored product that color intensity is proportional to the amount of protein in the sample was evaluated at 500 nm. Bovine serum albumin was used as a standard for determination of protein levels.

In the course of the study, each biochemical experiment was replicated independently in triplicate for each serum sample and kidney homogenate.

### Statistical Analysis

All experimental data were analyzed using GraphPad Prism 9.0.5 (GraphPad Software, San Diego, CA, USA). Results were expressed as mean ± standard deviation (SD). Group comparisons were performed using one-way ANOVA followed by Tukey’s post-hoc test. Statistical significance was set at *p* < 0.05.

## Results

**Fig. 2:**
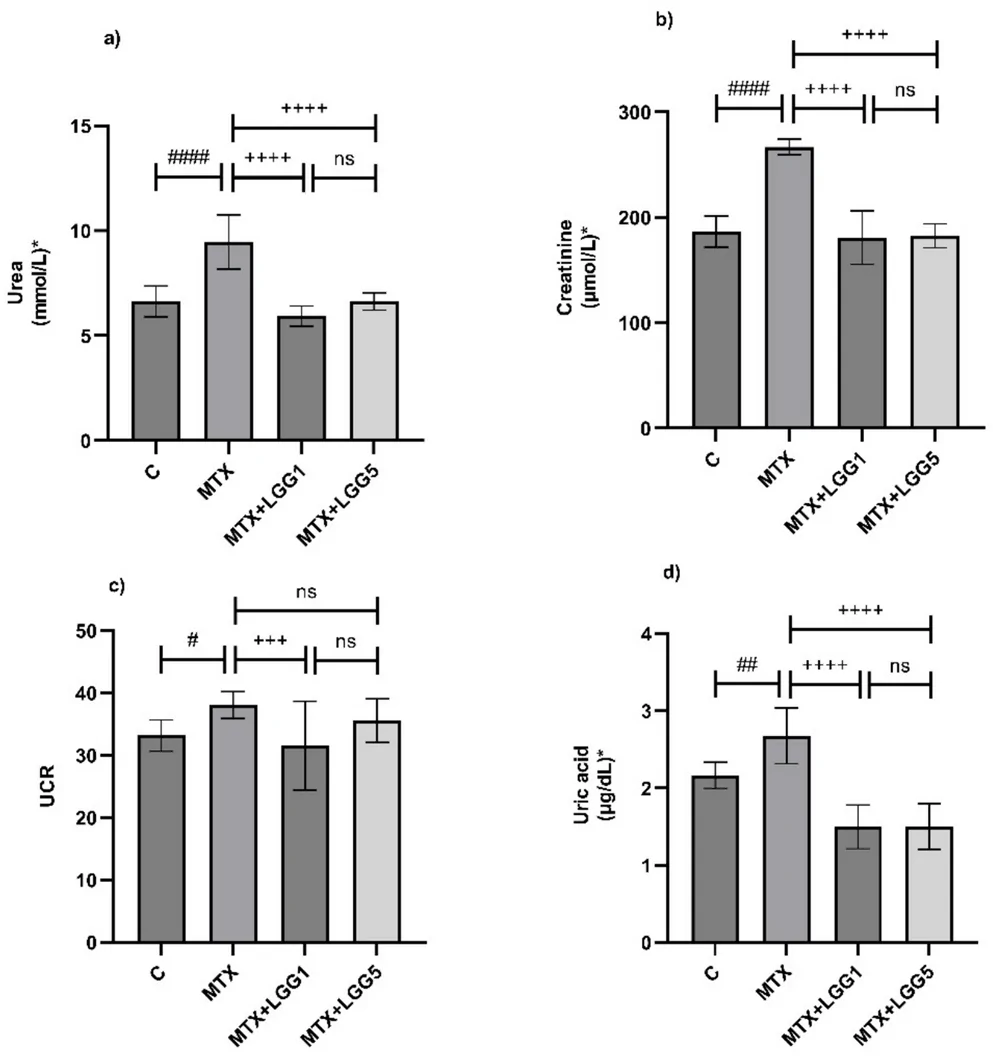
Serum urea, creatinine, UCR and uric acid levels of the control and experimental groups. All data were expressed as Mean ± SD. (**a**) Urea, (**b**) Creatinine, (**c**) UCR, and (**d**) Uric acid. C: Control, MTX: Methotrexate, MTX+LGG1: Methotrexate + *L. rhamnosus* (low dose), MTX+LGG5: Methotrexate + *L. rhamnosus* (high dose), UCR: Urea/Creatinine ratio, ns: Not significant. ####*p* < 0.0001, ##*p* < 0.01, #*p* < 0.05 versus control group, ^++++^*p* < 0.0001, ^+++^*p* < 0.001 versus MTX group

The evaluation of direct renal functions involved the assessment of urea, creatinine, UCR, and uric acid levels in serum. The statistical evaluations and mean assessment of urea, creatinine, UCR, and uric acid levels in the serum of rats for each group are presented in Fig. 2(a-d). MTX administration caused a pronounced elevation in urea (*p* < 0.0001), creatinine (*p* < 0.0001), UCR (*p* < 0.05), and uric acid (*p* < 0.01) levels compared with control group. LGG-treated groups (MTX+LGG1 and/or MTX+LGG5) exhibited significantly lower urea (*p* < 0.0001), creatinine (*p* < 0.0001), UCR (*p* < 0.001 for LGG1), and uric acid (*p* < 0.0001) concentrations compared with the MTX group. Conversely, no significant changes were detected between the MTX+LGG1 and MTX+LGG5 groups (Fig. 2a-d). Overall, MTX caused marked deterioration in renal functional markers, whereas LGG supplementation—particularly at lower doses—alleviated these MTX-induced changes.

**Fig. 3:**
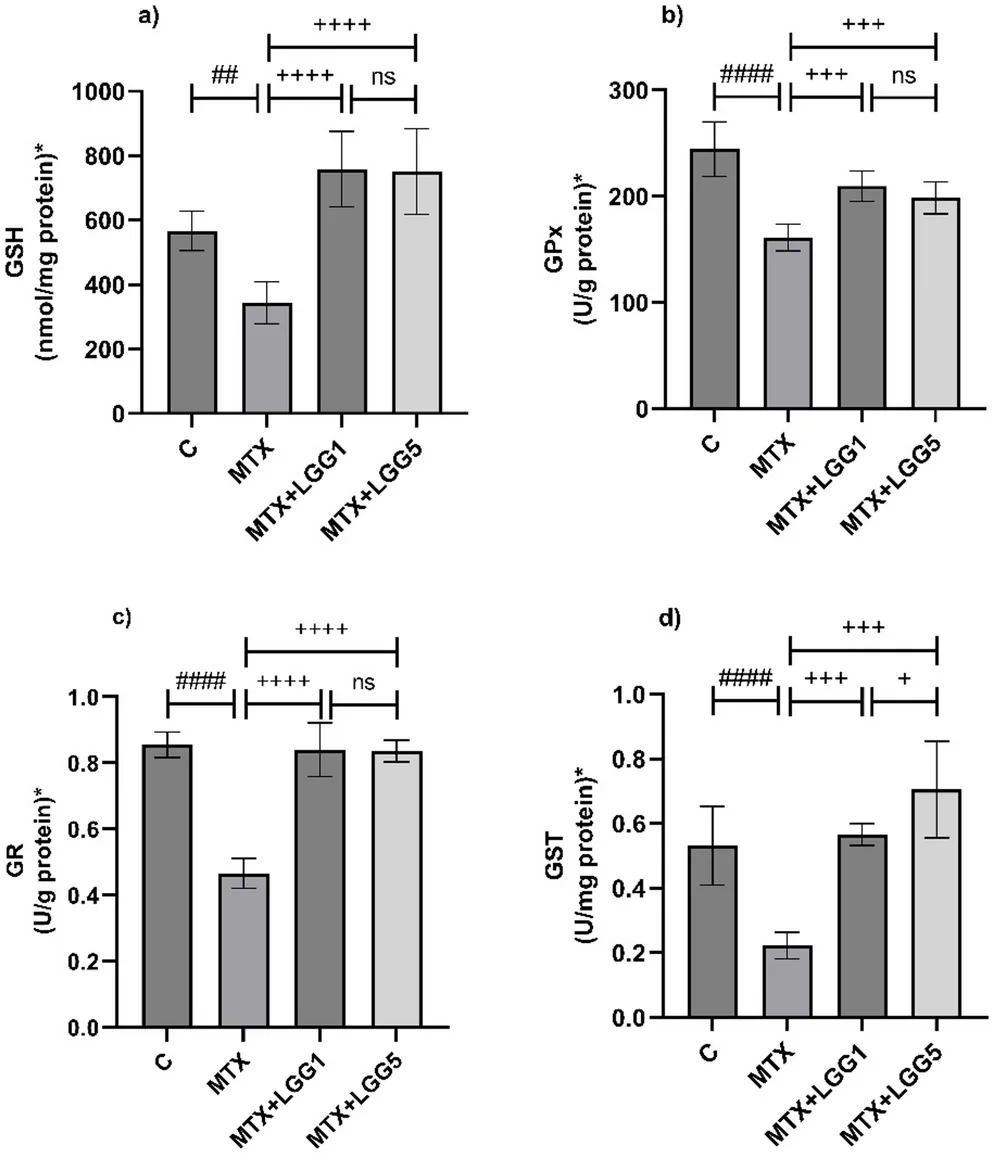
Kidney GSH levels and glutathione-related enzyme activities of the control and experimental groups. All data were expressed as Mean ± SD. (**a**) Reduced glutatione (GSH) levels, (**b**) Glutathione peroxidase (GPx), (**c**) Glutathione reductase (GR), and (**d**) Glutathione-S-transferase (GST) activities of the control and experimental groups. C: Control, MTX: Methotrexate, MTX+LGG1: Methotrexate + *L. rhamnosus* (low dose), MTX+LGG5: Methotrexate + *L. rhamnosus* (high dose), ns: Not significant. ####*p* < 0.0001 versus control group, ^++++^*p* < 0.0001 versus MTX group, ^++^*p* < 0.1 versus MTX+LGG1 group

The data showing GSH levels and glutathione-related enzymatic activities in the kidney tissue homogenates of the experimental and control groups of rats are presented in Fig. 3(a-d). Compared with the control group, MTX administration caused a marked decrease in GSH levels (*p* < 0.01) and GPx, GR, and GST activities (*p* < 0.0001), indicating weakened non-enzymatic and enzymatic antioxidant capacities. Both LGG-treated groups (MTX+LGG1 and MTX+LGG5) exhibited a significant recovery of these parameters compared to the MTX group (*p* < 0.0001 and *p* < 0.001). However, examining the MTX groups given LGG1 and LGG5 revealed a significant increase in GST activity (*p* < 0.05), unlike GPx and GR activities (Fig. 3a-d).

**Fig. 4:**
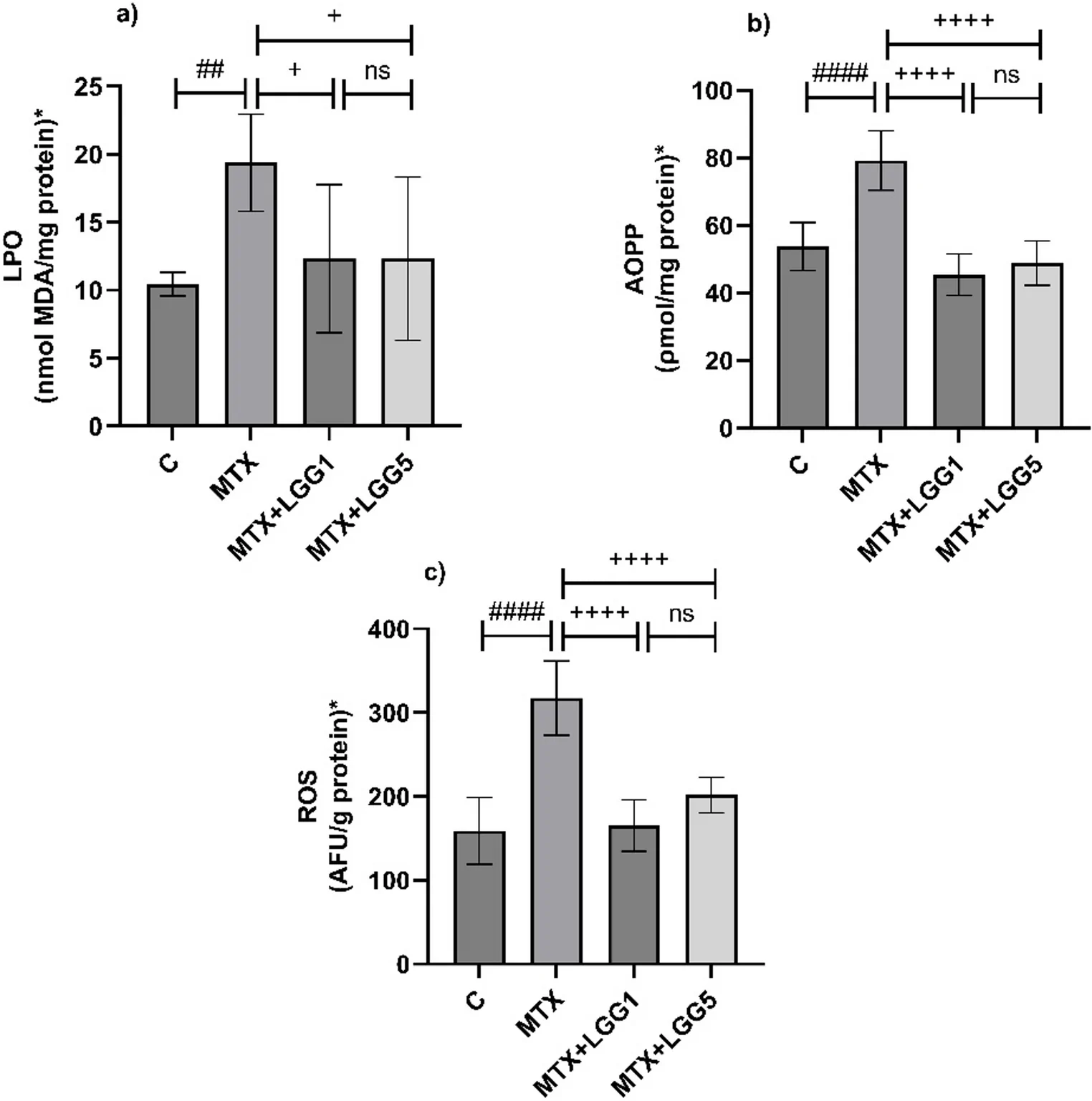
Kideny LPO, AOPP, and ROS levels of the control and experimental groups. All data were expressed as Mean ± SD. (**a**) Lipid peroxidation (LPO) levels, (**b**) Advanced oxidation protein products (AOPP) levels, (**c**) Reactive oxygen species (ROS) levels. C: Control, MTX: Methotrexate, MTX+LGG1: Methotrexate + *L. rhamnosus* (low dose), MTX+LGG5: Methotrexate + *L. rhamnosus* (high dose), ns: Not significant, AFU: Arbitrary fluorescence unit, MDA: Malondialdehyde. ##*p* < 0.01, ####*p* < 0.0001 versus control group, ^+^*p* < 0.05, ^++++^*p* < 0.0001 versus MTX group

The LPO, AOPP, and ROS levels were depicted in Fig. 4(a-c). According to Fig. 4, LPO, AOPP, and ROS levels were significantly higher in the MTX group (*p* < 0.01 and *p* < 0.0001, respectively) than in the control group. Conversely, LGG supplementation at both doses caused a notable decrease in LPO, AOPP, and ROS levels compared with the MTX group, suggesting enhanced membrane lipid recovery, reduced protein oxidation and improved ROS-scavenging potential of LGG (*p* < 0.05 and *p* < 0.0001, respectively). When compared to MTX group, no substantial difference was observed between the two LGG doses (Fig. 4a-c).

**Fig. 5:**
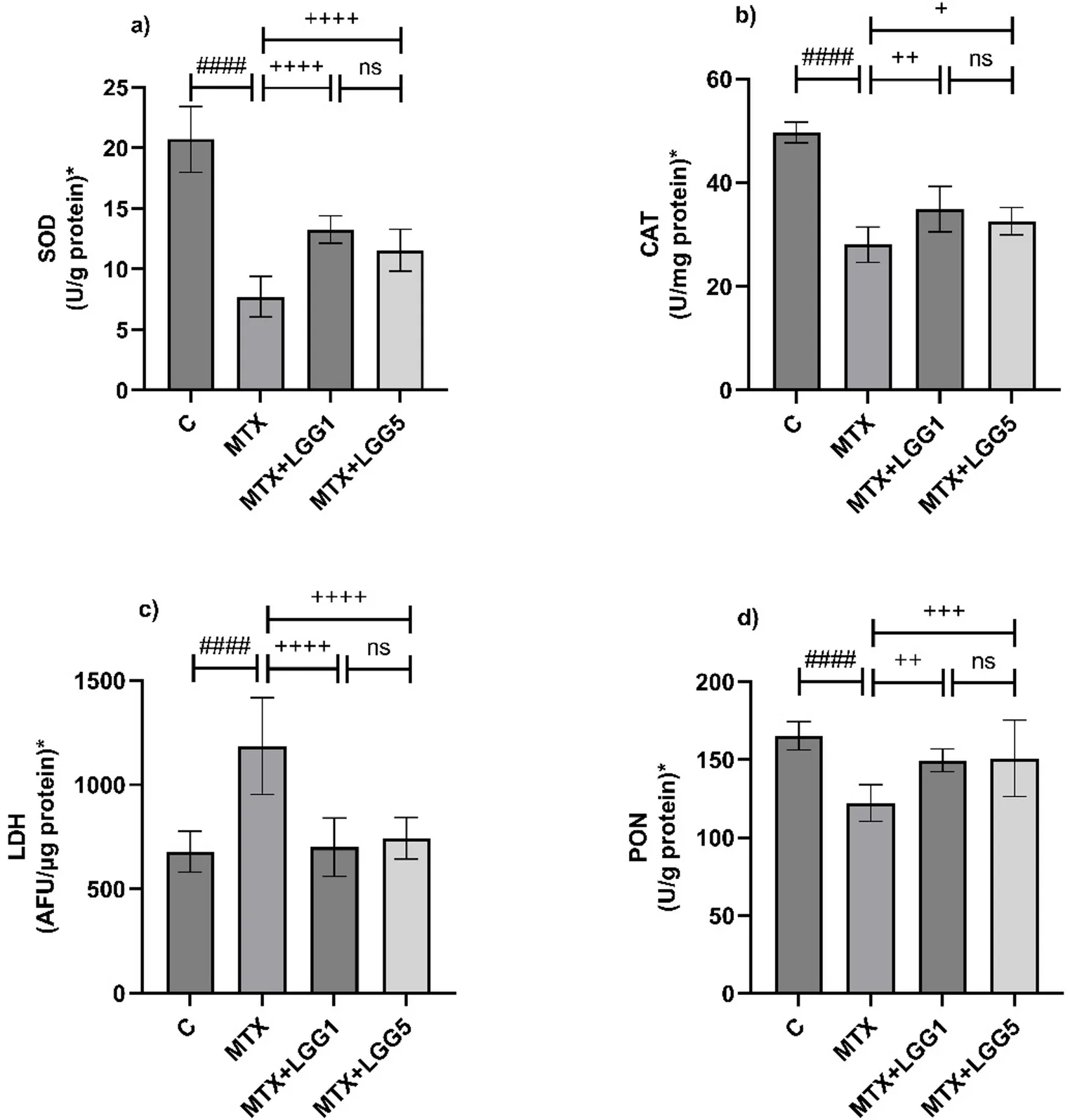
Kidney SOD, CAT, LDH, and PON activities of the control and experimental groups. All data were expressed as Mean ± SD. (**a**) Superoxide dismutase (SOD), (**b**) Catalase (CAT), (**c**) Lactate dehydrogenase (LDH), and (**d**) Paraoxonase (PON) activities of the control and experimental groups. C: Control, MTX: Methotrexate, MTX+LGG1: Methotrexate + *L. rhamnosus* (low dose), MTX+LGG5: Methotrexate + *L. rhamnosus* (high dose), ns: Not significant. AFU: Arbitrary fluorescence unit. ####*p* < 0.0001 versus control group, ^++++^*p* < 0.0001, ^++^*p* < 0.01, ^+^*p* < 0.05, ^+++^*p* < 0.001 versus MTX group

The findings of the first-line antioxidant defense markers SOD and CAT, LDH and PON activities are illustrated in Fig. 5(a-d). After a single dose of MTX administration to healthy rats, notable reductions of the SOD, CAT, and PON activities (*p* < 0.0001), while remarkable elevation of LDH activity was observed (*p* < 0.0001). In contrast, a significant improvement was recorded in SOD (*p* < 0.0001), CAT (*p* < 0.01 and *p* < 0.05, respectively), LDH (*p* < 0.0001), and PON (*p* < 0.01 and *p* < 0.001, respectively) activities when LGG1 and LGG5 supplemented with MTX group. However, no statistically significant difference was observed between the LGG1 and LGG5 groups, suggesting that both doses exerted comparable protection (Fig. 5a-d).

**Fig. 6:**
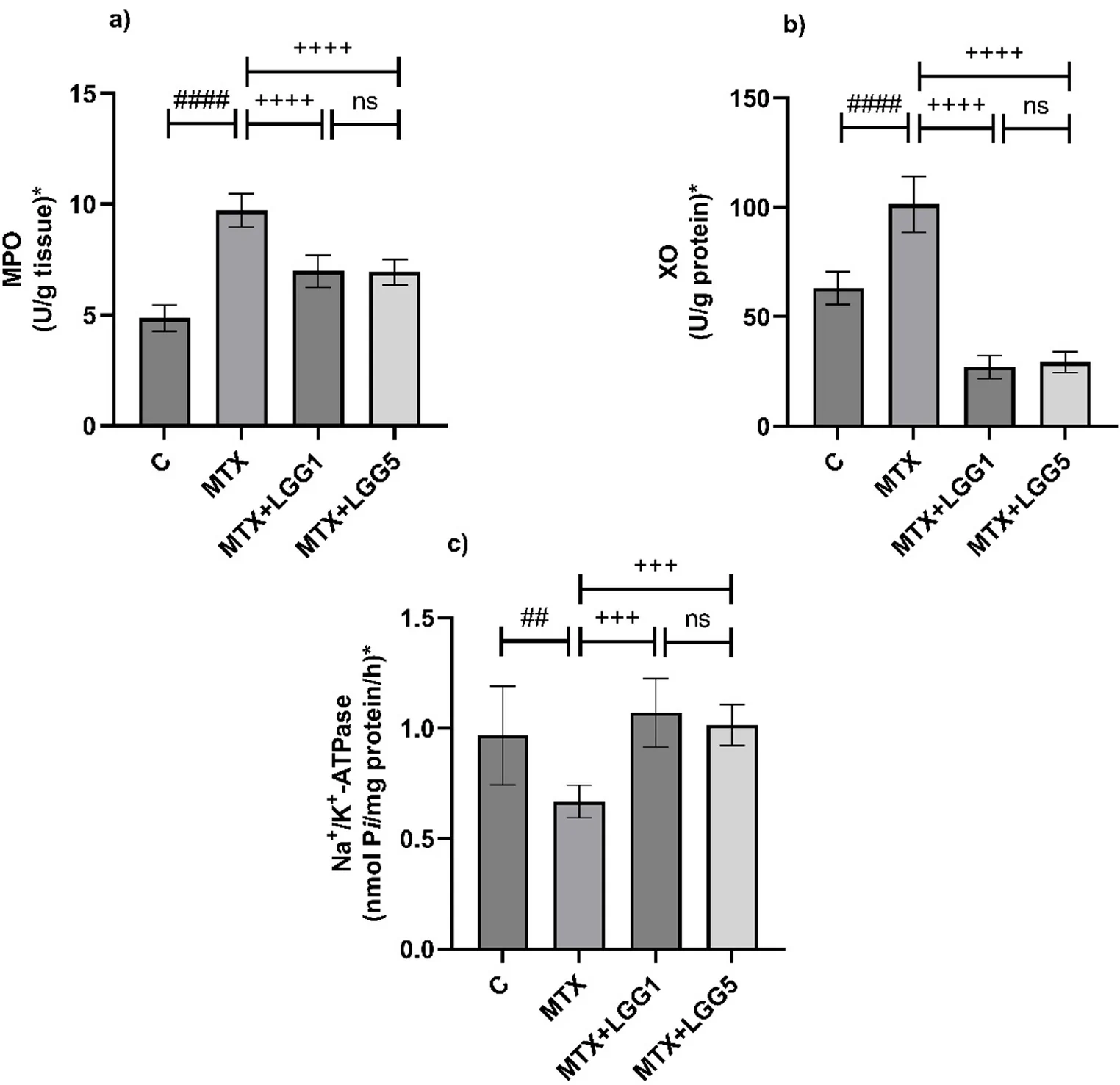
Kidney MPO, XO, and Na^+^/K^+^-ATPase activities of the control and experimental groups. All data were expressed as Mean ± SD. (**a**) Myeloperoxidase (MPO), (**b**) Xanthine oxidase (XO), and (**c**) Sodium/potassium ATPase (Na^+^/K^+^-ATPase) activities of the control and experimental groups. C: Control, MTX: Methotrexate, MTX+LGG1: Methotrexate + *L. rhamnosus* (low dose), MTX+LGG5: Methotrexate + *L. rhamnosus* (high dose), ns: Not significant. *P*_*i*_: Inorganic phosphate. ####*p* < 0.0001, ##*p* < 0.01 versus control group, ^++++^*p* < 0.0001, ^+++^*p* < 0.001 versus MTX group

Figure 6(a-c) shows the effects of MTX and LGG supplementation on MPO, XO, and Na^+^/K^+^-ATPase activities. Compared with the control group, MTX administration caused significant elevations in MPO (*p* < 0.0001) and XO (*p* < 0.0001) activities, indicating enhanced cellular/tissue injury and neutrophil activation. On the other hand, following MTX treatment Na^+^/K^+^-ATPase activity remarkably reduced (*p* < 0.01). In contrast to effects of MTX, administration of LGG at low and high dose to MTX given group led to remarkable decline in both MPO and XO activities (*p* < 0.0001) but a notable rise in the Na^+^/K^+^-ATPase activity (*p* < 0.001), indicating a clear protective effect against MTX-induced renal injury. However, no significant changes were detected between the MTX+LGG1 and MTX+LGG5 groups, suggesting that these have the same effect (Fig. 6a-c).

**Fig. 7:**
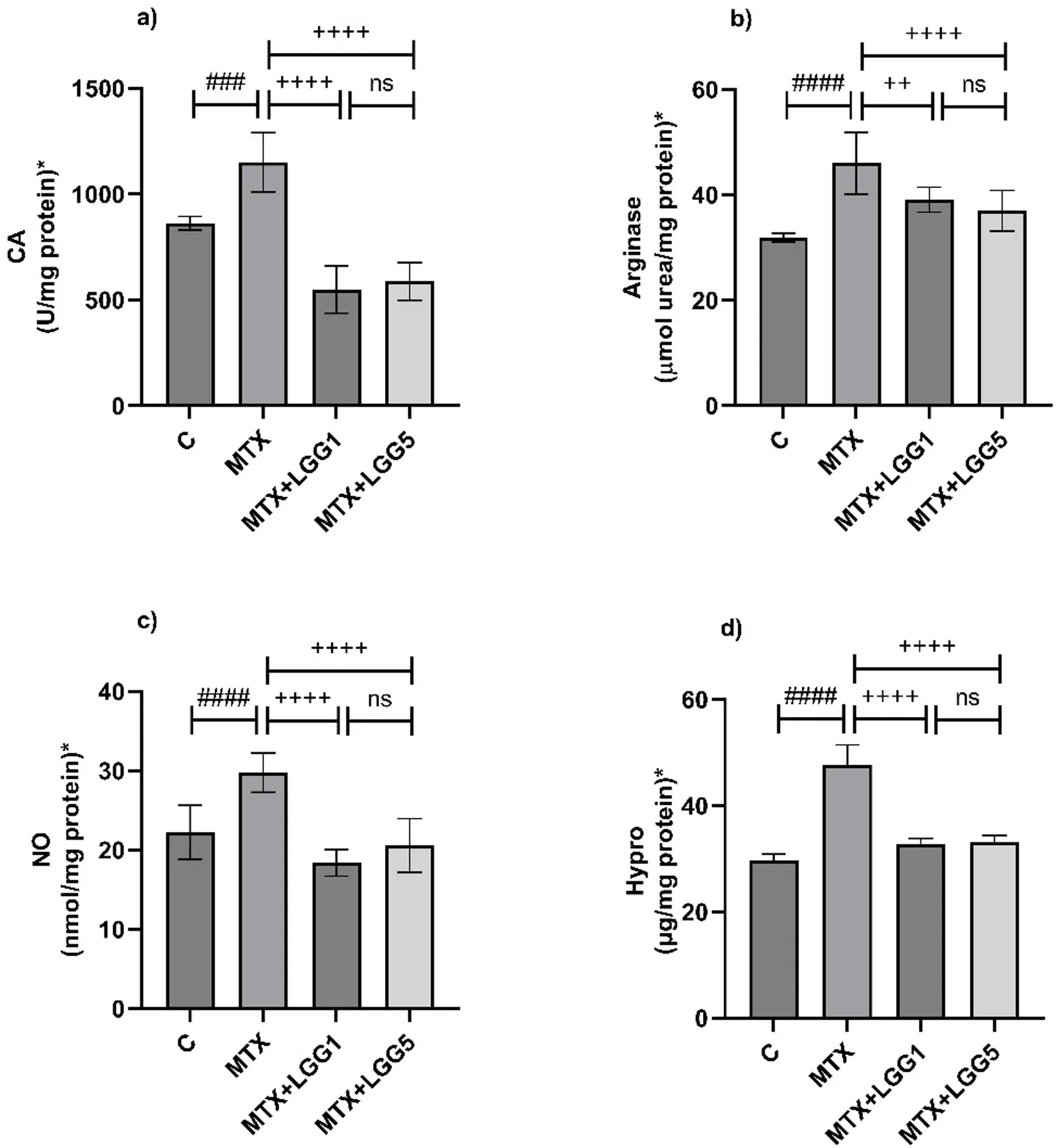
Kidney CA and arginase activities, NO and Hypro levels of the control and experimental groups. All data were expressed as Mean ± SD. (**a**) Carbonic anhydrase (CA) and (**b**) Arginase activities, and (**c**) Nitric oxide (NO) and (**d**) Hydroxyproline (Hypro) levels of the control and experimental groups. C: Control, MTX: Methotrexate, MTX+LGG1: Methotrexate + *L. rhamnosus* (low dose), MTX+LGG5: Methotrexate + *L. rhamnosus* (high dose), ns: Not significant. ###*p* < 0.001, ####*p* < 0.0001 versus control group, ^++++^*p* < 0.0001, ^++^*p* < 0.01 versus MTX group

The data showing activities of CA and arginase enzymes, and the levels of NO and Hypro of all groups are illustrated in Fig. 7(a-d). As seen in Fig. 7., a significant difference was shown in the activities of CA (*p* < 0.001) and arginase (*p* < 0.0001) enzymes and NO (*p* < 0.0001) and Hypro (*p* < 0.0001) levels in MTX group when compared to control rats. Co-treatment with LGG at both doses significantly reverted all these alterations of four markers compared with the MTX group (*p* < 0.0001 and *p* < 0.01). However, no statistically significant difference was observed between the LGG1 and LGG5 groups, suggesting that both doses exerted comparable protection (Fig. 7a-d).

## Discussion

MTX is widely used in the treatment of several major autoimmune disorders, including cancer, rheumatoid arthritis, and psoriasis, and is recognized as a potent cytotoxic agent [4]. Clinically, MTX is administered at high doses in malignant diseases and at lower doses for non-malignant autoimmune and inflammatory disorders [3]. Despite its therapeutic benefits, prolonged MTX administration is associated with adverse effects in multiple tissues, most notably the liver and kidneys, but also in the bone, testis, and lung [38]. Because more than 90% of MTX is excreted through the kidneys, it is considered a nephrotoxic compound [39]. The nephrotoxicity induced by MTX has been attributed to its ability to trigger OS, impair antioxidant defense systems, and elevate pro-inflammatory cytokines that contribute to cell death [40].

Recent evidence highlights the growing recognition of probiotic bacteria as potent modulators of oxidative pathways. Probiotics (*Lactobacillus* strains, such as *L. rhamnosus* GG.) exert their antioxidant effects through several mechanisms, including metal ion chelation, activation of endogenous antioxidant defense systems, regulation of ROS-producing enzymes [41]. In the present study, LGG administration was associated with improvements in MTX-induced biochemical and enzymatic alterations. However, the underlying mechanisms cannot be definitively established from the current data.

In this study, we investigated the potential protective effects of LGG administered orally at low (1 × 10⁹ CFU/day) and high (5 × 10⁹ CFU/day) doses for five consecutive days against MTX-induced renal injury following a single intraperitoneal dose of MTX (20 mg/kg). The evaluation was based on biochemical indicators of kidney function.

Urea represents the final product of protein metabolism and is synthesized in the liver through the detoxification of ammonia via the ornithine cycle. Impaired renal function reduces the elimination of urea, leading to elevated serum urea a well-recognized indicator of reduced glomerular filtration or tubular dysfunction [42]. Serum creatinine remains one of the most widely accepted markers of renal function and is central to the diagnosis of AKI [43]. Uric acid, the end-product of purine metabolism, possesses antioxidant capacity; however, its excessive accumulation enhances OS. Under normal physiological conditions, uric acid is efficiently cleared by the kidneys, while impaired glomerular filtration or tubular secretion increases its serum levels [44]. When taken together, serum urea, creatinine, and uric acid levels are among the most frequently used biochemical markers for assessing kidney function. In addition to these parameters, the urea/creatinine ratio (UCR) may be regarded as an indirect indicator of glomerular filtration rate (GFR), reflecting the kidneys’ capacity to eliminate metabolic waste products from the body [45]. Furthermore, an increase in UCR has been documented to result in a decrease in GFR due to elevated serum urea and/or creatinine levels.

In the present study, a significant rise in serum urea, creatinine, UCR, and uric acid levels was observed, reflecting acute impairment of renal filtration following MTX exposure. These outcomes align with previous reports showing that MTX caused elevations of serum urea [46] and creatinine concentrations in rats [47, 48]. The present study demonstrated that MTX administration also resulted in a substantial increase in UCR levels. A distinctive feature of creatinine is that, unlike urea, it cannot be reabsorbed by the kidneys [49], which may have contributed to the development of renal failure in MTX group. On the other hand, the observed increase in serum uric acid levels in response to MTX treatment can be associated with several potential mechanisms. These include the precipitation of MTX within the renal tubules, resulting in tubular damage and decreased filtration [50, 51]. In contrast, when low and/or high doses of LGG were administered concomitantly with MTX to rats, a statistically significant diminution was observed in the levels of these markers in the current study, suggesting that attenuation of oxidative damage in renal tissue. Similar findings have been reported in studies demonstrating the ability of probiotics to regulate serum renal biomarkers by reducing OS and inflammation through antioxidants and anti-inflammatory mechanisms [52–55].

The term ‘oxidative stress’ (OS) is generally defined as an imbalance between pro-oxidants and antioxidants. This occurs when the body’s non-enzymatic and enzymatic antioxidant defense systems are unable to neutralize excessively reactive oxygen and nitrogen species (ROS and RNS, respectively) [56]. The equilibrium between reactive species and antioxidant mechanisms, supported primarily by reduced and oxidized glutathione (GSH/GSSG), is tightly regulated [57].

GSH, one of the major non-enzymatic antioxidants in mammalian cells, protects against free radicals both directly and as a cofactor for antioxidant and detoxification enzymes such as GPx, GR, and GST [58]. Almost all intracellular glutathione exists in its reduced form (GSH), with only a small fraction present as GSSG, and this ratio is maintained by synthesis, oxidation–reduction cycles, detoxification reactions, and intracellular transport [59]. GPx detoxifies peroxides through GSH oxidation to GSSG, and GSSG is subsequently reduced back to GSH by GR using NADPH. GST uses GSH as a cofactor in xenobiotic detoxification [60]. In the present study, MTX significantly reduced renal GSH levels and GPx, GR, and GST activities compared with the control group. These reductions may reflect impaired GSH synthesis and excessive ROS formation, resulting in increased GSH utilization and decreased in GSH-related enzyme activities [61]. These findings are in harmony with previous article demonstrating MTX-induced OS and tissue injury in organs such as the liver and kidneys [62]. Conversely, both low and high LGG doses restored these alterations of biomarkers in MTX-treated rats, suggesting that LGG suppresses ROS overproduction by enhancing antioxidant capacity. Similar observations have been reported for *Lactobacillus* strains that scavenge hydroxyl and superoxide radicals [63], as well as for strains shown to limit ROS generation and increase GSH under doxorubicin-induced OS [64]. Moreover, LGG reversed OS-related changes in GSH levels induced by bisphenol-A in rats [14].

ROS are generated during mitochondrial electron transport and various enzymatic reactions, and include both radical (e.g. superoxide, hydroxyl radical and NO) and non-radical [e.g. singlet oxygen, hydrogen peroxide and hypochlorous acid (HOCl)] species [65]. At physiological levels, ROS participate in signaling and host defense. However, ROS have been shown to have a detrimental effect on membrane lipids (e.g. polyunsaturated fatty acids) in cells and/or organelles, leading to an increase in MDA levels — a by-product of oxidative lipid metabolism. This process is known as lipid peroxidation (LPO) [66, 67]. MDA has been identified as a highly significant biomarker frequently used to determine LPO levels [68]. AOPPs formed by oxidative modification and cross-linking of plasma proteins (e.g., via chloramines and HOCl), are established biomarkers of protein oxidation and tissue injury in organs such as the liver and kidney [69, 70]. In this study, MTX markedly increased renal ROS and AOPP levels, while decreasing GSH and increasing MDA. This indicates a substantial oxidative burden on the kidneys. Our data supports the idea that MTX promotes free radical formation, triggers LPO and protein oxidation, and weakens antioxidant defenses. These findings are consistent with reports of MTX-induced ROS overproduction, OS and kidney damage in rats [71]. LGG (at both doses) significantly reversed the rises in LPO, ROS and AOPPs induced by MTX, which is in parallel with previous data showing that LGG and other probiotics reduce OS markers, improve redox balance and normalize renal function parameters [72]. The effects of LGG administration attenuated changes in these parameters, suggesting a potent cytoprotective effect likely mediated through reinforcement of endogenous antioxidant systems and limitation of ROS-producing processes.

The primary enzymatic defense system (SOD, CAT, and glutathione-related enzymes) attempts to mitigate the harmful effects of excessive ROS production triggered by MTX or other exogenous agents; therefore, their activities often decrease under OS conditions [56]. SOD converts superoxide radicals into H₂O₂, which is then degraded to water by CAT [73]. In our study, it was established that the activities of these enzymes, which constitute the primary line of antioxidant defense in the kidney tissues of the MTX-treated group, were significantly diminished in comparison with the control group. Considering that MTX is taken up from circulation and is eliminated by the kidneys more than 90% of the time [74], it can be concluded that MTX is nephrotoxic and that its effects may also lead to changes in the relevant enzyme activities. This decline accompanied by GSH-related enzyme activities likely reflects MTX-induced ROS overproduction and cellular inability to compensate for the acute oxidative shift. More so, reduced GSH and elevated MDA further support OS development. Similar changes in antioxidant enzymes have been reported in MTX-induced hepatic and renal injury [75]. Importantly, LGG at both doses restored antioxidant enzyme activities and increased renal GSH, indicating that LGG stimulates both host and bacterial antioxidant defenses. Previous research showing probiotic-mediated enhancement of antioxidant enzymes support these findings [73].

LDH is released into the extracellular space when plasma membrane integrity is compromised; thus, increased LDH activity is widely regarded as a sensitive marker of necrosis and OS-related cell damage, particularly in chemotherapy-induced tissue toxicity [76]. PON is a calcium-dependent esterase associated with HDL that hydrolyzes lipid peroxides, thereby preventing lipoprotein oxidation and contributing to antioxidant defense, particularly in the liver and kidney [77]. This study found that MTX administration markedly increased renal LDH activity, but also notably decreased renal PON activity. This occurred due to the formation of aberrant ROS and increased LPO, indicating changes in membrane lipid composition and increased membrane permeability. Similar reductions in plasma PON activity following MTX treatment have been attributed to enhanced OS [78]. In contrast, both LGG-treated groups exhibited a significant decrease in LDH and PON activities compared with the MTX group, thus highlighting a protective role for LGG on the kidneys. This finding suggests that LGG may reduce LPO products and also indirectly enhance the antioxidant capacity associated with HDL and PON1, thereby contributing to the restoration of oxidative balance in kidney tissue. These findings agree with previous reports showing that LGG attenuates OS-induced cellular injury and decreases LDH activity in alcohol-induced intestinal and hepatic damage [79].

MPO, a heme-containing enzyme released by neutrophils and monocytes, uses hydrogen peroxide to produce strong oxidants such as HOCl, serving as a key mediator of innate immunity but also a driver of oxidative and nitrosative stress [80, 81]. XO, the rate-limiting enzyme of purine catabolism, catalyzes the oxidation of hypoxanthine and xanthine to uric acid, generating superoxide and hydrogen peroxide as by-products and thus contributing substantially to OS [82]. Na^+^/K^+^-ATPase is a membrane-bound ion pump that converts ATP energy into Na⁺ and K⁺ gradients, thereby maintaining transmembrane potential, ion balance, and osmotic stability, especially in the kidney [83]. In the present study, MTX significantly increased both XO and MPO activities but also declined Na^+^/K^+^-ATPase activities in renal tissue. This upregulation is consistent with reports that MTX disrupts mitochondrial function, elevates ROS production, and suppresses antioxidant defenses [40]. Similar reductions in renal Na^+^/K^+^-ATPase activity under MTX-induced OS conditions have been reported [84]. LGG treatment led to a substantial reduction in XO and MPO activities, while concurrently elevating Na^+^/K^+^-ATPase activities in renal tissue. These findings suggest that probiotic supplementation can mitigate the oxidative burden induced by MTX, thereby restoring redox balance. This, in turn, can contribute to the preservation of membrane integrity and ionic homeostasis through the antioxidant and anti-inflammatory properties of probiotics. Although probiotics may influence renal injury through gut-derived inflammatory and oxidative pathways, we did not directly assess microbiota composition, intestinal permeability, endotoxin levels, or gut-derived inflammatory mediators. Therefore, involvement of the gut–kidney axis should be considered a possible explanation rather than a directly demonstrated mechanism. Present findings observed the current study align with studies demonstrating that LGG enhances renal function and reduces OS markers in models of kidney injury, including those involving the gut–kidney axis [65, 85]. Furthermore, LGG has been shown to improve renal function and decrease creatinine and urea levels in models of chronic kidney disease [86], suggesting a broader nephroprotective role that extends beyond the stabilization of ion pumps.

A key metalloenzyme, CA, catalyzes the reversible hydration of CO₂ to bicarbonate and protons, thereby regulating pH, ion transport, and urine buffering, particularly in proximal tubular cells [87]. Arginase catalyzes the conversion of L-arginine to L-ornithine and urea [88]. Increased arginase activity can limit the availability of the substrate for nitric oxide synthase (NOS), thereby indirectly modulating NO production [89]. The present study showed that MTX treatment led to a significant increase in renal CA activity, which may reflect a compensatory response to MTX-induced acid–base disturbances and ionic imbalance. High-dose MTX has been shown to disrupt acid–base equilibrium and cause renal damage, while CA inhibition can mitigate these effects by alkalinizing the urine [90, 91]. Furthermore, MTX treatment was found to significantly increase both renal arginase activity and NO levels. These findings suggest that MTX may alter L-arginine metabolism, elevate serum urea, and disrupt redox balance, while simultaneously inducing iNOS and enhancing NO production [92]. Although increased arginase activity might theoretically limit NO synthesis, strong iNOS induction under inflammatory conditions can override this effect, resulting in elevated total NO metabolites [93, 94]. LGG supplementation significantly reduced CA activity toward control levels, although there is no in vivo evidence yet, that LGG directly modulates renal CA. Neverthless, LGG itself encodes the α-CA enzyme [91], and its antioxidant actions may indirectly normalize CA activity by stabilizing redox and ionic homeostasis [95]. However, since CA expression, isoenzyme-specific activity, and related signaling pathways were not examined, this finding should be interpreted as a biochemical association rather than direct evidence of LGG-mediated modulation. More so, LGG at both doses significantly reduced arginase activity and NO levels compared with the MTX group, suggesting that LGG mitigates OS and inflammation and normalizes L-arginine/NO metabolism. These observations agree with reports that probiotics modulate arginase expression and preserve NO bioavailability [96].

Hypro, a major component of collagen, is a biochemical marker of extracellular matrix (ECM) turnover and fibrosis in renal tissue [97, 98]. In this study, MTX significantly increased renal Hypro levels, indicating enhanced collagen deposition. Similar increases have been associated with fibrogenic processes in MTX-induced hepatic injury and other toxin-related liver fibrosis, where elevated Hypro correlated with heightened MDA, reduced GSH and antioxidant enzyme activities [99, 100]. In the present study, LGG supplementation significantly reduced Hypro levels, suggesting that LGG limits MTX-induced ECM disruption, preventing excessive collagen accumulation. This may have been likely achieved by attenuating OS and OS-related key metabolic pathways.

The present study should be interpreted as an acute experimental model of MTX-induced renal toxicity. Since MTX was administered as a single dose and the animals were sacrificed on day 6, the findings mainly reflect short-term biochemical and enzymatic changes. Therefore, these results do not establish the long-term therapeutic efficacy of LGG in chronic MTX nephrotoxicity. On the other hand, the present study is subject to certain limitations. Firstly, histopathological confirmation would not be confirmed. It is suggested that future research may provide further insights into microbiota-mediated gut-kidney interactions, thereby facilitating a more comprehensive understanding of the effects of LGG on mitochondrial function, apoptosis, and inflammatory signaling pathways. Secondly, the actual viable CFU administered during the experiment was not independently confirmed. Future studies should include microbiological verification of probiotic viability throughout the intervention period. It is anticipated that these studies will elucidate the systemic protective mechanisms of LGG. In order to evaluate the reliability and therapeutic potential of LGG in chronic MTX treatment, further studies using chronic or repeated-dose MTX models and longer follow-up periods evaluating different LGG doses in both animal models and human subjects should be conducted. Furthermore, the investigation of combinations of LGG with other probiotic strains may reveal possible synergistic effects on renal tissue.

## Conclusion

In conclusion, we demonstrated that a single nephrotoxic dose of MTX induced marked functional, biochemical, and oxidative alterations in rat renal tissue, whereas oral administration of LGG at both doses conferred significant protective effects. LGG improved classical renal function markers (urea, creatinine, UCR, and uric acid), restored antioxidant defenses (GSH and related enzymes), attenuated lipid peroxidation and protein oxidation, and reduced markers of membrane damage, and nitro-oxidative stress. These findings suggest that LGG attenuated short-term biochemical and enzymatic alterations associated with acute MTX-induced renal toxicity in rats. Further studies are required to determine whether these protective effects persist in chronic models and whether they have clinical relevance.

## Data Availability

The findings of this study are available from the corresponding author upon reasonable request.
